# A New Role for Vitamin D: The Enhancement of Oncolytic Viral Therapy in Pancreatic Cancer

**DOI:** 10.3390/biomedicines6040104

**Published:** 2018-11-05

**Authors:** Christopher J. LaRocca, Susanne G. Warner

**Affiliations:** Department of Surgery, City of Hope National Medical Center, Duarte, CA 91010, USA; clarocca@coh.org

**Keywords:** oncolytic virus, virotherapy, vitamin D, paricalcitol, pancreatic cancer

## Abstract

Oncolytic viruses have emerged as a novel class of anti-cancer therapeutics with one virus already receiving United States Food and Drug Administration (FDA) approval (talimogene laherparepvec) and many others undergoing testing in clinical trials. These viruses have direct lytic effects on tumor cells as well as immunomodulatory functions to increase inflammatory cell infiltrates in the tumor microenvironment. Despite all of the advances in cancer care, pancreatic cancer remains a highly lethal malignancy. One of the main barriers to successful systemic treatment of the disease is the fibrotic tumor stroma, as the unique extracellular matrix creates an environment that promotes tumor growth and is resistant to chemotherapy and other anti-cancer agents. The pleiotropic effects of Vitamin D have been widely studied, but recent research has now demonstrated it to be an effective agent in modulating pancreatic cancer stroma to facilitate the enhanced delivery of cytotoxic chemotherapy and immunogenicity in response to treatment. This review will explore the combination of Vitamin D with oncolytic viruses and how this novel application of Vitamin D’s ability to modulate pancreatic tumor stroma may result in a potential mechanism for increasing the efficacy of oncolytic virotherapy in pancreatic cancer.

## 1. Introduction

Oncolytic viruses are emerging therapeutics in the cancer treatment armamentarium. They are naturally occurring or genetically modified viruses that infect, replicate in, and kill cancer cells while minimizing damage to surrounding normal cells [1]. Multiple viral backbones are currently employed in vector development and include adenoviruses, reoviruses, herpesviruses, vaccina viruses, and coxsackieviruses [2]. Oncolytic viruses can be designed to selectively replicate in cancer cells through the use of a tumor-specific promoter or through key deletions in viral genomes [3,4,5]. Furthermore, many viral backbones are capable of accepting transgene insertions that can be used to facilitate real-time imaging (e.g., sodium iodide symporter [NIS]) to monitor viral infections or used to locally deliver a high concentration of a particular cytokine to augment the oncolytic effect (e.g., interferon alpha and granulocyte-macrophage colony-stimulating factor [GM-CSF]) [6,7,8]. The last decade has seen advances in vector design and development, which has culminated in the FDA’s approval of the first oncolytic virus for use in humans (talimogene laherparepvec [T-Vec]) [9]. Spurred on by the success of T-Vec, many other oncolytic viruses are entering into clinical trials as monotherapy agents or as part of a combination therapy regimen [2]. Despite promise in clinical settings, it is clear that for most malignancies a cure will not be achieved with an oncolytic virus as a monotherapy treatment. Oncolytic vectors have demonstrated synergy with chemotherapy and radiation, and many new studies have shown promising effects when these viruses are combined with immunotherapies [10,11]. In particular, the combination of oncolytic viruses and immune checkpoint inhibitors is an active area of investigation with promising initial results [12,13,14,15]. The future of oncolytic virotherapy will be tied to combination therapy regimens where the unique aspects of the individual components can work in concert to generate a more effective anti-cancer regimen.

When compared to many other solid tumors, the outcomes for pancreatic cancer patients remain poor [16]. In the United States in 2015, it was the fourth most common cause of cancer death among both men and women [16]. The overall five-year survival rate for all pancreatic cancers is approximately 7% [17]. Even for those patients able to undergo surgical resection, the five-year survival rates reported from experienced centers are still below 30% [18]. Modern chemotherapeutic regimens have resulted in modest survival improvements [19,20], but novel therapeutic regimens are needed for pancreatic cancers. One of the main challenges to successful therapy has been overcoming the pancreatic tumor stroma. A dense fibrotic reaction and altered extra-cellular matrix (ECM) creates a situation that is conducive to growth of the tumor [21]. Vitamin D has shown particular promise as a stromal modulator in pancreatic cancer. As Sherman et al. have established, the vitamin D receptor (VDR) expressed in the pancreatic cancer stroma can be bound by VDR-ligands to reduce inflammation and fibrosis in the tumor stroma, which results in increased delivery of cytotoxic therapies to the tumor [22]. As a consequence of the aforementioned findings, many new clinical trial protocols have incorporated Vitamin D or one of its analogs into pancreatic cancer treatment regimens.

This review will explore the nature of Vitamin D and its pleiotropic effects, with particular attention to treatment of pancreatic cancer, and will further explore the notion of enhanced oncolytic viral efficacy following Vitamin D treatment.

## 2. The Basics of Vitamin D

Vitamin D refers to a group of secosteroid molecules of which calcitriol [1,25(OH)_2_D3] is the biologically active form [23]. Calcitriol binds to the VDR, which is a member of the nuclear hormone receptor superfamily [24]. The liganded VDR then goes on to form a heterodimer with the Retinoic X Receptor (RXR) to exert transcriptional regulatory functions on target genes [25]. The VDR influences over 700 genes and it is estimated that Vitamin D and its effectors influence up to 3% of the human genome [26]. The VDR is expressed (to varying degrees) on most nucleated cells in the human body with particularly high concentrations in intestinal epithelia, renal tubules, parathyroid and pituitary tissue, pancreas, skin, immune system cells, and bone [27,28,29]. With VDR expression so widespread throughout the human body, it is no wonder that the Vitamin D system has such pleiotropic effects including anti-proliferative and anti-inflammatory effects, pro-differentiation, apoptosis, and the inhibition of angiogenesis [28,30]. Vitamin D’s main role is classically described in calcium and phosphate homeostasis, but it has also been implicated in the regulation of the cardiovascular and immune systems as well as numerous facets of cancer biology [25].

Vitamin D’s relationship to cancer has been widely studied on both epidemiologic and cellular levels. In the 1980s, researchers highlighted the association between geographic areas with reduced exposure to natural light (a surrogate for Vitamin D levels) and an increased incidence of colorectal cancers [31]. These findings prompted further analysis into the role that Vitamin D deficiency may play in cancer risk and if Vitamin D supplementation could be used in cancer prevention [32]. Similarly, one of the first reports of Vitamin D’s specific anti-cancer effects demonstrated that calcitriol inhibited the growth of in vitro melanoma cells [33]. In that same year, calcitriol was shown to also promote the differentiation of murine myeloid leukemia cells towards the macrophage lineage [34]. Since those early reports, Vitamin D and its analogs have been studied in many other solid malignancies including breast, prostate, and pancreatic cancers [35].

## 3. Vitamin D and the Stroma of Pancreatic Cancer

Within the pancreatic tumor stroma, the pancreatic epithelial cells, cancer-associated fibroblasts, and pancreas stellate cells (PSC) create a microenvironment that is pro-tumor and anti-chemotherapeutic [36]. The PSCs are of particular interest because they are normally in a quiescent state, but can be activated during times of injury and stress and transform into myofibroblast-like cells [37]. They then continue to secrete ECM proteins that lead to the fibrotic stromal reaction that is characteristic of pancreatic cancer [38]. Furthermore, activated PSCs have been shown to enhance pancreatic cancer cell activation through the secretion of factors such as platelet-derived growth factor and insulin-like growth factor 1 [22,39]. As the desmoplastic reaction continues and the tumor progresses from pre-invasive to invasive disease, the immune cell infiltrate in the tumor microenvironment undergoes marked changes [40]. Tumor-associated macrophages, myeloid-derived suppressor cells (MDSC), and regulatory T cells are increasingly present and serve to create a locally immunosuppressive environment [41]. MDSCs achieve this effect, in part, by inhibiting the activation of CD 4^+^ and CD 8^+^ T cells as well as decreasing NK (natural killer) cell cytotoxicity [42]. Additionally, matrix metalloproteinases (e.g., MMP-8 and MMP-9) derived from tumor associated neutrophils (TAN) further contribute to the immunosuppressive nature of the tumor microenvironment [43]. The aforementioned dense fibrotic reaction and alterations in the tumor immune milieu have been recognized as major contributors to the treatment failures of many current-generation pancreatic cancer therapeutics [21,36,40].

Given their role in promoting the fibrotic tumor stroma and secretion of tumor-promoting factors, activated PSCs represent an attractive therapeutic target. The Evans research group has analyzed the effect of the VDR on PSCs and potential impact of VDR-mediated stromal remodeling in the treatment of pancreatic cancer. Importantly, they showed that there are sustained levels of the VDR in both normal and cancer-associated PSCs, which was previously not known to be the case for the exocrine pancreas [22,44]. Using the VDR ligand (calcipotriol), they also established how the activated VDR can regulate the activated PSCs on a transcriptional level. For example, they demonstrated calcipotriol-dependent inhibition of numerous activation and cancer signatures including regulators of angiogenesis such as Thsb1 [22]. Using the genetically engineered KPC pancreatic cancer mouse model [45] and combination therapy with a VDR-ligand and gemcitabine, they showed decreased tumor volumes with the combination therapy (when compared to either monotherapy), augmented tumor vasculature, increased intratumoral gemcitabine concentrations, and altered gene expression including an induction of Fabp4 (a PSC quiescence marker) [22]. The use of a VDR-ligand to reprogram the pancreatic cancer stroma was a major breakthrough for pancreatic cancer research and has since prompted Vitamin D analogs to be included in numerous ongoing clinical trials (Table 1).

## 4. Oncolytic Viruses, Tumor Stroma, and Barriers to Successful Therapy 

A distinguishing characteristic of oncolytic viruses is their ability to selectively replicate and amplify within tumors, something that no other cancer therapeutic can do [46]. Assuming that the virus can successfully infect a tumor cell, one of the key components to its potency and efficacy is related to its ability to spread to and infect other cells within the tumor. Mathematical models analyzing intratumoral injections of oncolytic viruses have determined that optimal tumor eradication occurs with widespread and diffuse viral distribution upon infection [47]. However, one of the inherent challenges with intratumoral delivery of viruses is that the viral spread is limited and often does not extend far beyond the site of injection [48,49]. One of the key intrinsic physical barriers that limit the spread of an oncolytic vector is the tumor ECM [48]. In pancreatic cancer and other solid tumors, there is an altered ECM that is composed of growth factors, cytokines, tumor-associated vasculature, immune cells, and fibroblasts [21]. In addition to the physical barrier created by the dense fibrotic stroma, there is also an increased interstitial fluid pressure that further decreases an oncolytic virus’ ability to spread throughout the tumor [50]. Therefore, the number and rate of injections, along with the volume of infusion, can greatly affect the initial viral distribution within a tumor following direct injection [51].

Numerous approaches have been employed to increase viral spread within the tumor including degradation of the ECM, increasing viral production and release, and induction of apoptosis [48,52]. Given the fibrotic nature of the pancreatic cancer tumor stroma, strategies for degrading or modulating the ECM are of particular interest to many researchers. Kuriyama et al. analyzed the use of proteases as a pretreatment in subcutaneous xenografts of glioblastoma tumors prior to the injection of a recombinant non-replicating adenovirus [53]. Pretreatment consisted of trypsin or collagenase/dispase with intratumoral viral injection the following day and subsequent analysis of transgene expression (beta-galactosidase). Trypsin resulted in increased gene expression, but an equally important finding of these experiments was that the dose and timing of the pretreatment affected the level of viral transduction (high doses of protease actually decreased transduction efficiency) [53]. In addition, other research groups have explored the use of hyaluronidase, matrix metalloproteinases, and relaxin to overcome physical limitations to oncolytic viral therapy imposed by the ECM of particular tumors [54,55,56].

Targeting the ECM has proven to be an effective strategy for improving viral spread throughout the tumor, but the effectiveness of any particular enzymatic agent is relative to the specific makeup of the ECM in the tumor for which it is being used [48]. Additionally, important consideration must be given to the manner in which the therapeutic will be delivered, namely as a transgene encoded by the oncolytic virus or given as a separate injection. If given as an injection, optimization of the timing and dosing will be critical to maximize viral spread within any given tumor’s stroma.

While the ECM is one of the main barriers to the success of intratumoral viral delivery, there are multiple additional factors that affect the success of systemic, intravenous delivery of oncolytic vectors. Neutralizing antibodies, the complement cascade, sequestration in the liver/spleen, and sub-optimal extravasation from tumor-associated blood vessels must be taken into consideration [2,57]. For example, many patients have a high rate of neutralizing antibodies due to previous vaccinations and environmental exposures [58]. Following the second and additional exposures to an oncolytic virus, there is often an augmented immune response which has necessitated the development of multiple mechanisms to limit viral neutralization [46]. Serotype switching, host immunosuppression, viral shielding with polymer coating or PEGylation, and the use of carrier molecules to deliver viruses directly to the tumor bed are examples of currently employed strategies [59,60,61].

Despite the limitations to both intratumoral and intravenous delivery of oncolytic viruses, multiple vectors have been evaluated in clinical trials for pancreatic cancer patients or are currently undergoing testing (Table 2) [62,63,64,65,66].

## 5. Vitamin D Use in Oncolytic Virotherapy

Vitamin D has been implicated as one of the substances that may help to regulate the body’s response to viral infection (especially to enveloped viruses) [67]. In some instances, it has been associated with a negative effect on viral replication via the induction of antimicrobial peptides including cathelicidin (LL-37) and human beta defensin 2 [67,68]. Human cathelicidin has been shown to negatively affect and decrease the viral replication of certain rhinovirus, lentivirus, vaccinia virus, herpes simplex virus (HSV-1), and adenoviruses [69,70,71,72]. Interestingly and importantly, upon treatment with LL-37, the degree of viral replication was not consistent across different adenovirus (Ad) serotypes. LL-37 demonstrated a statistically significant reduction in the titer of Ad 19, while there was only a non-significant trend towards decreased replication in other serotypes including Ad 3, Ad 5, and Ad 8 [71]. Through the induction of gene expression of certain antimicrobial peptides, Vitamin D may have an effect on viral replication, but the degree of such an effect is specific to the type of virus and the serotypes within a given viral family.

Vitamin D has also been employed as part of a vector system designed to selectively replicate in prostate and renal cell cancers. Hsieh et al. reported a replication-competent adenoviral vector (based on Ad 5) employing a human osteocalcin promoter (used to drive the adenoviral *E1A* and *E1B* genes) which contains a Vitamin D responsive element [73]. Here, the aforementioned virus selectively replicated in osteocalcin-expressing prostate cancer cells, and there was over a five-fold increase in viral replication when combined with Vitamin D [73]. Importantly, there was no evidence of decreased viral replication with the addition of Vitamin D to the cell cultures [73]. Additionally, Vitamin D has also been used to regulate the same human osteocalcin promoter-driven oncolytic adenovirus when it was loaded into human mesenchymal stem cells and used to treat renal cell carcinoma [74].

Vitamin D’s effect on viral replication is variable, but the aforementioned experiments using an adenovirus employing a human osteocalcin promoter (with a Vitamin D responsive element) provide evidence that successful viral infection and replication is not precluded by the presence of Vitamin D in culture. Future experiments will be needed to determine the vector system best suited to combination therapy with Vitamin D, as well as timing and dosing of the individual components.

## 6. Checkpoint Inhibition, Oncolytic Immunotherapy, and Pancreatic Cancer

As our understanding of pancreatic cancer’s unique immunosuppressive and pro-tumor microenvironment has grown, so has the potential to incorporate novel immunomodulatory therapeutics into the treatment regimens. Historically, pancreatic ductal adenocarcinomas have not responded well to monotherapy with immune checkpoint inhibitors when tested in clinical trials [75,76] and this is at least in part due to the poor immunogencity and decreased immune cell infiltrates of these tumors [77]. Vitamin D functions not only as a stromal reprogrammer, but it also modulates the immune system and plays a role in T cell activation, which may alter the tumor microenvironment to make checkpoint inhibitors more efficacious [78,79]. Multiple clinical trials (Table 1) are currently exploring the combination of immune checkpoint inhibitors and Vitamin D analogs (with and without other chemotherapeutics) for the treatment of pancreatic cancer.

Similarly, the field of oncolytic immunotherapy is evolving as the understanding of the post-viral infection immune milieu increases. Viral inoculation causes an influx of antigen-presenting cells, activated T cells, natural killer cells, and cytokines into the tumor microenvironment [11]. This type of modulation can transform an immunologically “cold” tumor with few immune cells into a “hot” tumor with an abundance of circulating immune effectors [80,81]. Furthermore, oncolytic virus infections can cause increased levels of PD-L1 (programmed death receptor ligand 1) expression on tumor cells [82,83]. Currently, there is one clinical trial that incorporated oncolytic virotherapy and checkpoint inhibition for patients with metastatic pancreatic ductal adenocarcinoma (NCT02620423). Here, intravenous Reolysin [Pelareorep] (a live, replication competent reovirus) was tested in combination with pembrolizumab in a phase Ib study that demonstrated a tolerable side effect profile and anti-tumor activity with three out of five patients having a partial response or stable disease [84].

Oncolytic viruses can serve as a type of immunotherapy to transform the tumor microenvironment and augment the local immune lymphocytic infiltrate. Immunologically “hot” tumors with upregulated expression of immune markers are more responsive to therapy with checkpoint inhibitors and serve as a rationale for combining these agents with oncolytic viruses [85]. Vitamin D will likely work in concert with these viruses to further prime the tumor to be more susceptible to novel combination therapies with checkpoint inhibitors.

## 7. Conclusions

Oncolytic virotherapy is a valuable member of the cancer treatment armamentarium. Despite the growth in this field and other cancer therapeutics, the five-year overall survival rate for pancreatic cancer still remains below ten percent. Vitamin D’s role in modulating the pancreatic cancer stroma has inspired a wave of clinical trials incorporating Vitamin D analogs into the treatment regimen. VDR-stromal reprogramming weakens the capacity of the PSCs to support cancer growth via stromal remodeling, alters the PSC phenotype to a more quiescent nature, and ultimately reduces fibrotic content and increases tumor vasculature [11]. One of the biggest barriers to the spread of oncolytic viruses is the ECM and the fibrotic stroma of pancreatic tumors is particularly restricting. Through the aforementioned mechanisms, Vitamin D can increase the delivery of cytotoxic chemotherapeutics to the tumor cells to augment cancer-killing capabilities. The combination of Vitamin D and oncolytic virotherapy may be able to overcome the dense pancreatic cancer stroma to demonstrate the potential efficacy of this novel combination therapy. As one of Vitamin D’s pleiotropic effects is to modulate the immune system, it may have a role in sensitizing tumors to other immunotherapies such as checkpoint inhibitors. Future avenues of investigation should explore the potential in the combination of oncolytic viruses, Vitamin D, and checkpoint inhibitors.

## Figures and Tables

**Table 1:** Key pancreatic cancer clinical trials employing Vitamin D analogs.

Trial Identifier	Study Name	Study Phase	Study Drugs	Design	Disease Status	Estimated Enrollment
NCT 03331562	A SU2C Catalyst^®^ Randomized Phase II Trial of the PD1 Inhibitor Pembrolizumab With or Without a Vitamin D Receptor Agonist Paricalcitol in Patients with Stage IV Pancreatic Cancer Who Have Been Placed in Best Possible Response	II	Pembrolizumab, Paricalcitol	Randomized	Metastatic	24
NCT 03300921	A Phase Ib Pharmacodynamic Study of Neoadjuvant Paricalcitol in Resectable Pancreatic Cancer	Ib	Paricalcitol	Non-Randomized	Resectable	20
NCT 03519308	A Pilot Study of Perioperative Nivolumab and Paricalcitol to Target the Microenvironment in Resectable Pancreatic Cancer	I	Nivolumab, Nab-paclitaxel, Gemcitabine, Paricalcitol	Randomized	Resectable	20
NCT 03520790	Vitamin D Receptor Agonist Paricalcitol Plus Gemcitabine and Nab-paclitaxel in Patients with Metastatic Pancreatic Cancer	I/II	Gemcitabine, Nab-paclitaxel, Paricalcitol	Randomized	Metastatic	112
NCT 02030860	A Randomized Pilot/Pharmacodynamic/Genomic Study of Neoadjuvant Paricalcitol to Target the Microenvironment in Resectable Pancreatic Cancer	Pilot	Gemcitabine, Nab-paclitaxel, Paricalcitol	Randomized	Resectable	15
NCT 03415854	A Phase II Pilot Trial of Paclitaxel Protein Bound Plus Cisplatin Plus Gemcitabine and the Addition of Paricalcitol Upon Disease Progression in Patients with Previously Untreated Metastatic Pancreatic Ductal Adenocarcinoma (NABPLAGEMD)	II	Gemcitabine, Nab-paclitaxel, Cisplatin, Paricalcitol	Single Group	Metastatic	14
NCT 02754726	A Phase II Pilot Trial of Nivolumab + Albumin-Bound Paclitaxel + Paricalcitol + Cisplatin + Gemcitabine (NAPPCG) In Patients with Previously Untreated Metastatic Pancreatic Ductal Adenocarcinoma	II	Nivolumab, Nab-paclitaxel, Cisplatin, Gemcitabine, Paricalcitol	Single Group	Metastatic	10
NCT 03138720	A Phase II Study of Paclitaxel Protein Bound + Gemcitabine + Cisplatin + Paricalcitol as Pre-operative Treatment in Patients with Untreated Resectable, Borderline Resectable and Locally Advanced Adenocarcinoma of the Pancreas	II	Gemcitabine, Nab-paclitaxel, Cisplatin, Paricalcitol	Single Group	Resectable, Borderline Resectable and Locally Advanced	24
NCT 02930902	A Preoperative Phase 1B Study to Assess the Safety and the Immunological Effect of Pembrolizumab in Combination with Paricalcitol With or Without Chemotherapy in Patients with Resectable Pancreatic Cancer	Ib	Pembrolizumab, Paricalcitol, Gemcitabine, Nab-paclitaxel	Non-Randomized	Resectable	30

**Table 2:** Key oncolytic virotherapy clinical trials in pancreatic cancer.

Trial Identifier	Study Phase	Virus Type	Virus Name	Virus Dose, Schedule	Virus Route	Transgene/Modifications	Study Regimen	Status/Results
−	I/II	Adenovirus	ONYX-015	2 × 10^10^ – 2 × 10^11^ vp, multiple injection	IT	*E1B* deletion	Weekly viral injections (total of 8), gemcitabine given with last 4 doses	10 of 21 patients had a partial response or stable disease, PMID: 12576418
NCT 02705196	I/IIa	Adenovirus	LOAd703	5 × 10^10^ – 5 × 10^11^ vp, multiple injection	IT	TMZ-CD40L, 4-1BBL	Gemcitabine + nab-paclitaxel given in 28 day cycles, Virus injected on Day 15 of 1st cycle and every 2 weeks (6 doses total)	Recruiting
NCT 02045589	I	Adenovirus	VCN-01	1 × 10^11^ – 1 × 10^13^ vp, multiple injection	IT	human PH20 hyaluronidase	Viral injections (every 28 days, total of 3) alone or in combination with gemcitabine or nab-paclitaxel	Completed, no published results
NCT 03252808	I	Herpesvirus	HF-10	1 × 10^6^ – 1 × 10^7^ TCID_50_/mL, multiple injection	IT	Naturally lacking UL56 expression	Virus administered at 2 week intervals in conjunction with chemotherapy (gemcitabine, Nab-paclitaxel, or TS-1)	Recruiting
NCT 00402025	I	Herpesvirus	Talimogene laherparepvec (T-Vec)	10^4^ – 10^7^ pfu/mL, multiple injection	IT	GM-CSF	Virus injected every three weeks (3 doses total), dose-escalation study	Completed, no published results
NCT 02620423	Ib	Reovirus	Pelareorep (Reolysin)	4.5 × 10^10^ TCID_50_, multiple infusion	IV	-	Virus (Day 1, 2 of 21 day cycle), Chemotherapy [Gemcitabine, irinotecan, or 5-FU] (Day 1), Pembrolizumab (Day 8)	Completed, no published results
NCT 01280058	II	Reovirus	Pelareorep (Reolysin)	3 × 10^10^ TCID_50_, multiple infusion	IV	-	Carboplatin/paclitaxel (Day 1 of 21 day cycle), Virus (Day 1−5)	No improvement in PFS when compared to chemotherapy control arm, PMID: 27039845
NCT 02714374	Ib	Vaccinia Virus	GL-ONC1 (GLV-1h68)	1 × 10^9^ – 5 × 10^9^ pfu, single/multiple infusion(s)	IV	Renilla luciferase-Aequorea green fluorescent protein (RUC-GFP)	Single and multiple dose cohorts, multiple dose cohorts receive daily virus injection (5 total doses) prior to surgery	Active, not recruiting

vp: viral particle; pfu: plaque forming unit; IT: intratumoral; IV: intravenous; TCID_50_: 50% Tissue culture infectious dose; PFS: progression-free survival; GM-CSF: Granulocyte-macrophage colony-stimulating factor; TMZ: trimerized membrane-bound isoleucine zipper.
